# Snakebite Envenomation, Attitudes, and Behavior toward Snakes in Banten, Indonesia

**DOI:** 10.3390/ani12162051

**Published:** 2022-08-11

**Authors:** Linda T. Uyeda, Ardiantiono, Entang Iskandar, Aaron J. Wirsing, Randall C. Kyes

**Affiliations:** 1 School of Environmental and Forest Sciences, University of Washington, Seattle, WA 98195, USA; 2 Durrell Institute of Conservation and Ecology (DICE), School of Anthropology and Conservation, University of Kent, Canterbury CT2 7NZ, UK; 3 Primate Research Center, IPB University, Bogor 16680, Indonesia; 4 Departments of Psychology, Global Health, and Anthropology, Center for Global Field Study, Washington National Primate Research Center, University of Washington, Seattle, WA 98195, USA

**Keywords:** snakes, Indonesia, behavior, conservation, snakebite envenomation

## Abstract

**Simple Summary:**

Globally, snake populations are in decline, with conservation efforts hindered by negative attitudes. Meanwhile, snakebite envenomation has been recognized as a serious public health issue, particularly in rural areas where humans and snakes coexist. This study reports the results of a survey conducted in Banten, Indonesia, with the aim of exploring relationships between respondents’ experiences, attitudes towards snakes, and behaviors towards snake populations. Our results indicate that snakebite envenomation presents a real threat in our study area, and that venomous snakes are regarded as highly dangerous. Individuals who had heard of others experiencing venomous snake bites were more likely to want populations of venomous snakes to decrease, and those with negative attitudes towards snakes were also more likely to engage in anti-conservation (“try to kill”) behavior. Women were more fearful than men, and women and those with lower education levels were more negative toward non-venomous pythons, suggesting that tailoring snake conservation efforts to specific groups based on experiences, education level, and attitudes may increase effectiveness. We propose that greater community knowledge of snakes and increases in local resources and preparedness can also contribute to accomplishing both snake conservation and improved public safety through prevention of snakebite envenomation.

**Abstract:**

Snakes are commonly associated with feelings of anxiety or disgust, and snake conservation is often hindered by negative attitudes and perceptions. Although global snake populations are generally in decline, snakebite envenomation (SBE) continues to be recognized as a serious public health issue, particularly in rural areas of tropical and subtropical countries. Data on SBE, a neglected tropical disease, are lacking, and Indonesia, a hotspot of venomous snake diversity, has no snake bite reporting system. We analyzed 127 survey results in Banten, Indonesia with the aim of documenting SBE and exploring the relationships between respondents’ experiences, attitudes, and behaviors toward snakes. Nine percent of respondents had experienced SBE, and knowledge of SBE incidents was associated with negative attitudes toward snake populations, with negative attitudes toward snakes associated with a higher likelihood of anti-conservation behavior. Women were more fearful than men, and women and those with lower education levels were more negative toward pythons (*Malayopython reticulatus*), suggesting that increased knowledge may aid in snake conservation efforts. Universally negative risk beliefs and attitudes toward venomous snakes indicate a need to reduce the threat of SBE in our study area.

## 1. Introduction

Many factors determine human behavior toward wildlife, and encounters with different types of wildlife meet with varying human responses. Whereas certain groups of wildlife may be revered or appreciated for their beauty (e.g., [1]), others elicit anxiety or disgust. Snakes often fall into the latter category [2,3,4]. In an Australian survey of animal preferences, for example, snakes were listed as the least favorite animal by over half of the respondents, with such animals most commonly described as “cause harm” and “ugly” [5]. Such negative values toward snakes can translate to persecution and anti-conservation attitudes [6].

While the threat of snakes can be overestimated by some individuals who encounter them (e.g., [7]), snakebite envenomation (SBE) has been recognized as a serious public health issue, particularly in rural areas of tropical and subtropical countries [8,9,10]. In June 2017, the World Health Organization (WHO) formally listed SBE as a highest priority Neglected Tropical Disease [11], and in 2019, WHO launched a global “Strategy for the Prevention and Control of Snakebite Envenoming” [12]. Despite increasing awareness of the issue, data on SBE incidence in rural areas are deficient, and severe knowledge gaps hinder efforts to address prevention, mitigation, infrastructure, and antivenom shortages [13]. Indonesia, in particular, has no formal snake bite reporting system [14] and has experienced a historical lack of SBE documentation [10,15,16]. Meanwhile, southeast Asia has been identified as a hotspot of venomous snake diversity [17] and Indonesia, with over 10 million at-risk individuals, is noted as one of the most populous areas vulnerable to SBE [17].

Globally, snake populations are in decline [18], with popular trade species such as the reticulated python, *Malayopython reticulatus*, experiencing a high harvest burden [19], and venomous species such as Asian cobras (*Naja* spp.) predicted to experience negative impacts due to climate change and anthropogenic pressure [20]. Whether visceral responses, fear for public safety, or additional factors contribute to attitudes and behaviors toward snakes, an understanding of these influences is necessary to inform appropriate conservation actions. Greater knowledge of local challenges, snake experiences, and location-specific species distributions are also needed to support prevention and mitigation of SBE in rural areas. As interviews conducted through previous research revealed residents’ encounters with both venomous and non-venomous snakes in village areas of Banten, Indonesia, we returned to these locations to administer a questionnaire based on the concepts identified through our initial efforts. Here, we present the survey results from this component of our research on local attitudes, experiences, and responses regarding wildlife in these village areas. We provide descriptive information relating to experiences, attitudes, and responses to *M. reticulatus* and venomous snakes in our study area while also comparing responses between groups and genders and modeling attitudes and behavior toward snakes based on multiple explanatory variables.

In presenting this case study, we aim to explore the influence of demography and experiences on respondents’ attitudes toward snakes, and the relationship between local attitudes, fear, and behavior related to the conservation and management of snake populations. We also document instances of SBE in our study area and contribute knowledge of venomous snake encounters in a rural Indonesian area. Based on our results, location-specific recommendations on approaches toward snake conservation and SBE mitigation are discussed.

## 2. Materials and Methods

### 2.1. Study Area

**Figure 1:**
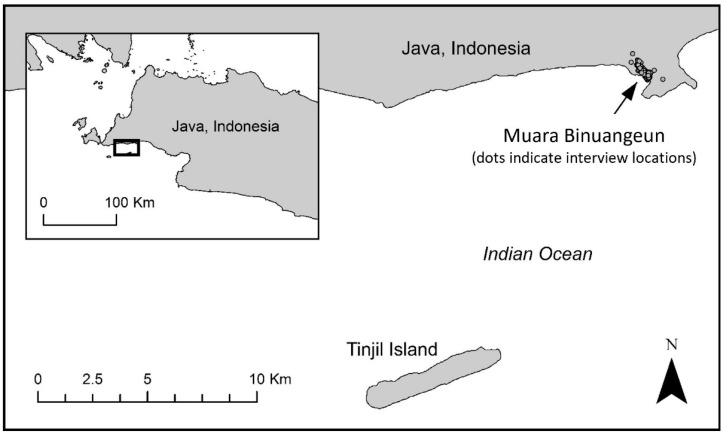
Map of the study area in Banten, Indonesia, including Tinjil Island, the village area of Muara Binuangeun, and locations where surveys were administered as individual in-person interviews. Inset shows the larger view, with a rectangle indicating the area of detail.

This study was conducted in Banten, Indonesia, with a focus on the mainland village area of Muara Binuangeun (MB; Figure 1), consisting of the villages of Muara Dua and Binuangeun. Participants with experience on nearby Tinjil Island, most of whom lived in MB or in neighboring villages, were also included in the study. The village area of MB was selected as an ideal location for evaluating local attitudes and perspectives toward snakes in areas where human activity and the ranges of numerous snake species overlap, as previous research had revealed local encounters with both venomous and non-venomous snakes [21].

Tinjil Island was established as a Natural Habitat Breeding Facility for Long-Tailed Macaques (*Macaca fascicularis*) in 1987 [22] and is largely undisturbed, consisting primarily of lowland secondary tropical rainforest and coastal vegetation. Although Tinjil Island has no permanent human inhabitants, it is staffed year-round with a small group (5–8 individuals at any given time) of caretakers who rotate off and on the island. The island is managed by the Primate Research Center of Bogor Agricultural University, and unauthorized killing or removal of flora and fauna from the island is prohibited. Snake species on Tinjil Island include *M. reticulatus*, the Indonesian bronze-back (*Dendrelaphis pictus*), and the venomous Javanese pit viper (*Trimeresurus puniceus*).

### 2.2. Sampling and Data Collection

This study utilized a two-stage approach. In the first phase, completed in August 2013, focus group and individual interviews were conducted, with the aim of identifying key concepts relating to human perceptions, attitudes, and experiences in communities where humans and the water monitor lizard, *Varanus salvator*, coexist (see [21]). All interviews were conducted by the first author with the assistance of a native Indonesian speaker conversant in both *bahasa Indonesia* (Indonesian) and *bahasa Sunda* (Sundanese). Interviewees were asked about their perceptions and knowledge in regard to local herpetofauna, with an initial focus on experiences related to *V. salvator*. Interview questions remained open-ended, with the aim of encouraging each participant to freely express their own perspectives and attitudes. In this way, our intention was to develop a grounded understanding of the themes most relevant to the participants themselves, rather than test specific hypotheses [23]. Interviews were carried out with the goal of attaining theoretical “saturation”, a point at which further interviews did not reveal significantly newer knowledge. Interview data were analyzed qualitatively through the process of theoretical coding [23], where emergent concepts from the interviews were grouped to identify themes reflecting the beliefs and attitudes of the participants, and to ground theory that was then used to develop a survey/questionnaire. This first stage of research was conducted with an exempt determination from the University of Washington Human Subjects Division (study #44076).

The second stage of the project, reported here, was carried out from 16 August 2014–26 September 2014, and entailed the administration of the survey instrument in the study area. As snake attitudes and encounters with snakes were mentioned by interview participants in the first stage of our research, the subsequent questionnaire contained questions designed to aid in a greater understanding of themes expressed by respondents in the initial research phase. The survey also included questions on demographic variables, and behavior toward snakes. Surveys were administered in person by the first and second authors, with the goal of obtaining a representative sample of Muara Dua residents. We attempted to interview an adult from every third household we encountered throughout the village, approaching potential respondents at their residences, providing a brief description of the purpose and format of the survey, and requesting participation. In the case of Tinjil Island staff, participants either completed the survey while working on the island or were met at their residences on the Javan mainland. With participants’ permission, GPS coordinates were taken from each interview location with a Garmin eTrex Vista HCx handheld GPS unit.

The survey component of our research received an exempt determination from the University of Washington Human Subjects Division (study #47655). Both stages of this project received approval from the Indonesian Ministry of Research and Technology, permit number 290/SIP/FRP/SM/VIII/2013.

Based on the first stage of interview data, *M. reticulatus* was reputed to be the only large (>2 m in length) non-venomous snake species in the study area. As smaller non-venomous snakes such as *D. pictus* were typically dismissed by respondents as inconsequential, we focused our survey questions relating to non-venomous snakes on *M. reticulatus* (“pythons”). We collected data on “venomous snakes” as a general category, as there are several known venomous snake species in our study area. Photos of local snake species were available as a reference for participants throughout the administration of the survey to ensure that responses regarding pythons and venomous snake species were accurately recorded.

Participants were asked their gender, age, education level, occupation, religion, and ethnic group. As Tinjil Island is associated with site-specific taboos that influence behaviors toward the island’s wildlife but are not recognized in the nearby village area of MB [21], survey participants were also asked whether they had ever worked on Tinjil Island.

Data on participants’ experiences with snakes were gathered by asking participants the number of times pythons/venomous snakes had been seen around their home in the last 12 months, if any problems had been associated with pythons or venomous snakes in the area around their home in the past 12 months, and if so, the nature of the problem. In addition, respondents were asked:

1. “How many times have you been injured (bitten) by venomous snakes in your life?”;
2. “How many times have you known/heard of people who have been bitten by venomous snakes?”

If an individual indicated that they had been bitten by a venomous snake, additional anecdotal information was recorded, such as the circumstances surrounding the experience and how the bite was treated. Respondents indicating that they had knowledge of a person being bitten by a venomous snake were asked whether the person was known to have died as a result of the bite.

Two attitudinal questions were asked in regard to both pythons and venomous snakes:

1. “How would you feel if you were to see a [python/venomous snake] in your neighborhood?” Respondents to this question were asked to select one answer from a five-point scale ranging from “enjoy seeing it” to “hate seeing it”;
2. “Given the choice, would you prefer the populations of [pythons/venomous snakes] in your neighborhood to increase, decrease, or stay the same?”

To assess risk beliefs and fear among respondents, participants were asked:

1. “How dangerous do you believe [pythons/venomous snakes] are to people?”

Respondents were offered four response choices: “not dangerous”, “somewhat dangerous”, “very dangerous”, and a “don’t know” option;

2. “How afraid are you of [pythons/venomous snakes]?”, also with four response choices, “not afraid”, “somewhat afraid”, “very afraid”, and “don’t know”.

Behaviors toward snakes/responses to snakes were measured by the following questions:

1. “What do you typically do when you see a [python/venomous snake] in your neighborhood?” with the response choices, “do not see it”, “nothing”, “chase it away”, “try to kill it”, “try to catch it to use or sell”, and “other”;
2. “In the past year, how many times have you killed a [python/venomous snake] in your neighborhood?” with the response choices of “never”, “1–3”, “4–7”, and “>7”.

### 2.3. Data Analysis

Chi-squared tests for homogeneity were conducted to compare responses between men and women and to identify differences in attitudes, risk beliefs, and behavior toward snakes among respondents. The Fisher exact test was used to compare the responses of men with experience on Tinjil Island with those of men with no experience on Tinjil Island. The paired sample sign test was used to compare responses from individuals with experiences on Tinjil Island regarding their own neighborhood with responses regarding Tinjil Island.

Variables were selected for model building by using the Spearman correlation test and the Chi-squared test for independence to evaluate the correlation between covariates. Covariates with no strong correlation (Spearman test, R < 0.7; X^2^, *p* > 0.05) were included in the model building process. Gender, education, experiences hearing about others bitten by venomous snakes, and personal experiences of SBE were evaluated as covariates for building an explanatory model of attitudes toward venomous snakes. Gender, age, education, and experiences seeing pythons were selected as covariates for building initial models of attitudes toward pythons. Other experiences related to pythons were not available, as we did not systematically collect corresponding data on whether individuals had been bitten or knew of others bitten by pythons. As reported occupations were highly gender specific (Table 1), occupation was not included as an additional variable in the model building process. Three covariates, consisting of attitudes of feeling when seeing snakes, preference for snake population trends, and fear, were used to evaluate potential models predicting behavior toward both pythons and venomous snakes.

For each individual model analysis, we performed the step-wise model building approach to construct a set of cumulative link models (CLMs; [24]), first starting with a null model (no covariates) and global model (models including all covariates), and then building subsequent models by excluding covariates with the least support based on *p*-values (higher *p*-value, lower support). Models were ranked using AICc [25], and model averaging of the top models (ΔAICc < 2) was performed. Model convergence was evaluated through parameter assessment accuracy to the global model of every model set [25]. All global models showed a reported error log-likelihood value below 10^−10^, which indicated accurate parameter estimates. All statistical analyses were performed in R version 4.02 [26].

## 3. Results

In total, 127 surveys were administered in person (71 male and 56 female), with interviews ranging from 18 min to 1 h and 51 min long. Survey interviews averaged 37 min (median was 35 min). All interviews were recorded with the participants’ permission, with the exception of four individuals who were interviewed but declined to be recorded. The demographic profile of the respondents (Table 1) shows a religiously homogeneous population (100% Muslim), with Sundanese as the predominant ethnic group (82%). The majority of the participants (53%) were aged 31–45, with 74% of the participants reporting an elementary school education or lower. The most commonly reported occupations were fisherman (25%) for men and housewife (28%) for women. In total, 31 individuals, all men (24% of the total participants), had current or previous experience working on Tinjil Island.

### 3.1. Reported Experiences

Only 12 individuals (9%) had seen a reticulated python in the area around their home in the past 12 months, but, in the same time period, 52 individuals (41%) had encountered a venomous snake near their home at least once. The majority of respondents did not report having experienced any problems associated with snakes around their homes over the most recent 12 months; only 2 individuals reported problems with pythons in that they ate their chickens, ducks, or eggs while 17 individuals (13%) stated that venomous snakes had been considered a problem because they could injure people. However, additional reported experiences indicated that SBE remained a concern for our study population; 3 respondents had actually been bitten by a venomous snake in the most recent year, and 12 individuals (9%) had been bitten by a venomous snake at some point in their lifetimes. Over half of interview participants (*n* = 69, 54%) stated that they knew of at least one person who had died as the result of SBE.

Anecdotal information gathered regarding experiences relating to SBE included six mentions of the efficacy of rubbing “kodok” (referring to local frog/toad species) skin or organs on the bite wound, and three individuals who noted the use of “daun daun” (traditional herbal medicine) in the treatment of venomous snake bites. Three respondents explained that they had gone to a medical doctor for treatment, with two of them specifically describing injections of antivenom. One antivenom recipient explained that as a child, she had been bitten by an “ular tanah” (Malayan pit viper, *Calloselasma rhodostoma*) when out at night relieving herself in a field (having had no toilet in her village home) but had been able to travel to a doctor straightaway for an injection of antivenom, recovering fully. However, many years later when her father had been bitten on the hand by the same species when working in a rice paddy, he was unable to obtain the proper treatment in time, and subsequently died. Two additional unrelated individuals also relayed that their fathers had died as a result of SBE.

Javan spitting cobra (*Naja sputatrix*), king cobra (*Ophiophagus hannah*), and kraits (*Bungarus* spp.) were additional venomous snake species mentioned as having been encountered in our study area.

### 3.2. Attitudes and Risk Beliefs

Attitudes toward seeing pythons were varied, with 21% (*n* = 27) reporting that they would either “enjoy seeing it” or were “interested in seeing it”, 33% (*n* = 42) selecting “don’t care”, and 45% (*n* = 57) stating that they “don’t like seeing it” or “hate seeing it”. Three of the respondents explained that their interest in seeing a python was for the sake of catching it to sell. Despite many seemingly ambivalent responses to seeing a python, 76% (*n* = 97) of individuals stated that if given a choice, they would like the number of pythons in their neighborhood to decrease.

Respondents expressed stronger negative attitudes toward venomous snakes, with 81% (*n* = 103) of the respondents either selecting “don’t like seeing it” or “hate seeing it” when asked how they would feel if they were to see one in their neighborhood, and 92% (*n* = 117) stating that given the choice, they would like populations of venomous snakes in their neighborhood to decrease.

Thirty-one individuals with experience on Tinjil Island were also asked whether they would prefer for populations of pythons and venomous snakes on Tinjil Island to increase, stay the same, or decrease. More respondents indicated that they would like populations of both pythons and venomous snakes on Tinjil Island to stay the same or increase as compared to populations in their own neighborhoods (paired-samples sign test, pythons *n* = 12, *p* < 0.001, d = 1.2; venomous snakes *n* = 17, *p* < 0.001, d = 3.15). The attitudes of men with experience on Tinjil Island did not differ from men without experience on Tinjil Island (*n* = 40) when comparing responses regarding preferred snake population trends for their own neighborhoods (Fisher exact test, *n* = 71: venomous snakes, *p* = 0.22; python, *p* = 0.33).

**Figure 2:**
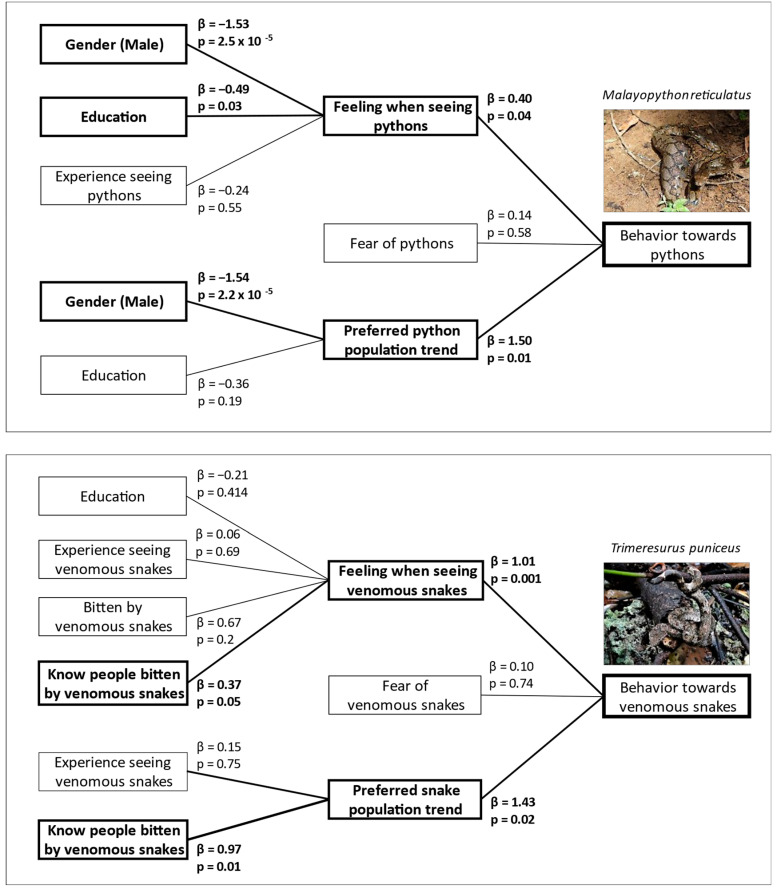
Cumulative link model diagram describing the explanatory variables of attitudes and behavior towards snakes based on model averaging of the top models with ΔAICc < 2. Variables listed in the left column were represented in the top performing models based on the initial model building process, which were then averaged to identify significant predictors for attitudes toward snakes. Attitudes, including “feeling when seeing snakes”, “fear of snakes”, and “preferred snake population trend”, were then used as covariates to evaluate potential models predicting behavior toward both pythons and venomous snakes. Model-averaged coefficients (β) represent the strength and direction of the predictors, with positive coefficients indicating more negative attitudes/behavior (e.g., hate seeing snakes/prefer snake populations to decrease/try to kill snakes). Significant predictors (*p* < 0.05) are highlighted in bold.

Model averaging of the top models (based on AICc ranking) revealed significant associations between gender and education level and respondents’ feelings upon encountering a python (Figure 2, Table 2). Female respondents were less likely to have positive feelings toward pythons than males, whereas respondents with higher education tended to enjoy seeing pythons. Conversely, feelings upon seeing a venomous snake were strongly associated with experiences of knowing someone bitten by snakes; those with knowledge of a higher number of individuals experiencing snake bites tended to report negative feelings toward snakes (i.e., “don’t like” or “hate” seeing snakes).

Our models revealed that gender again explained respondents’ preference for python population trends. More male respondents preferred python populations to be stable or increase compared to female respondents. Desire for populations of venomous snakes to go up or down were strongly explained by respondents’ experiences of hearing about others being bitten by venomous snakes; those who knew many bitten individuals tended to favor a decrease in venomous snake numbers. Models including the variable of age were not top performing in the initial model building process and were thus not included in the final model averaging analyses for feelings upon encountering a python and preferences for python population trends.

**Figure 3:**
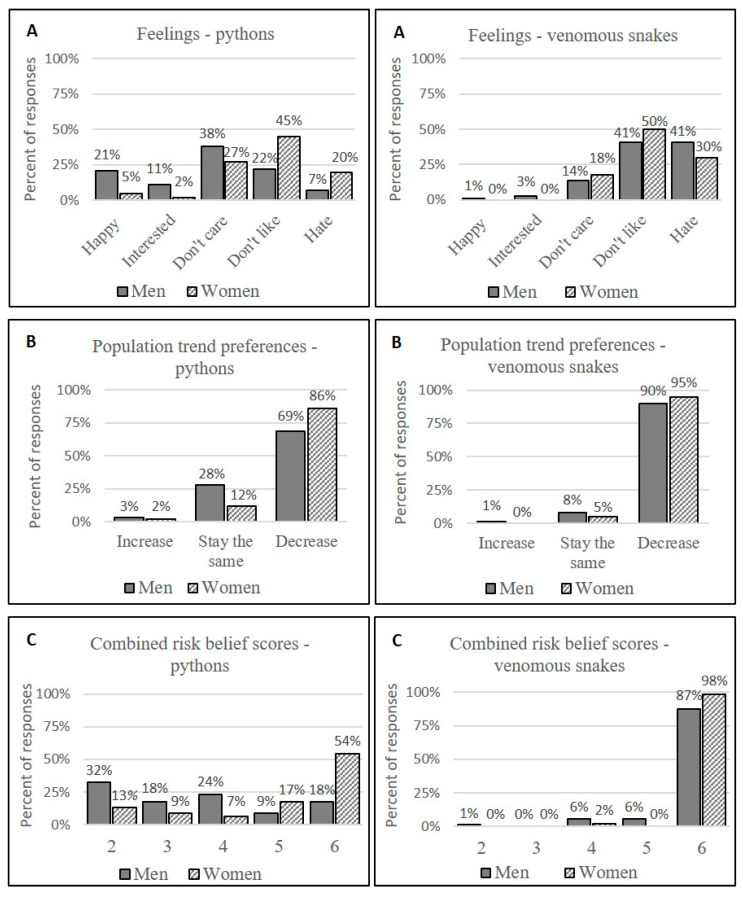
Attitudes towards snakes based on the responses of male and female survey participants (*n* = 71, men; *n* = 56, women) in Banten, Indonesia: (**A**) Feelings when seeing a python/venomous snake; (**B**) Preferred populations trends for pythons/venomous snakes in neighborhood areas; (**C**) Combined risk belief scores, consisting of coded answers to the questions “How dangerous do you believe pythons/venomous snakes are to people?” and “How afraid are you of pythons/venomous snakes?”, where 2 corresponds to a “not dangerous” (score of 1) plus “not afraid” (score of 1) response, and 6 corresponds to a “very dangerous” (score of 3) plus ”very afraid” (score of 3) response.

Although only 38% (*n* = 48) of the respondents considered pythons to be “very dangerous”, and 44% reported being “very afraid” of them, women were more likely to consider pythons “very dangerous” (X^2^ (3, *N* = 127) = 15.39, *p* = 0.001, V = 0.348) than men, and to characterize themselves as “very scared” (X^2^ (3, *N* = 127) = 30.24, *p* < 0.001, V = 0.488) when asked how afraid they were of pythons (Figure 3). Nine women (16%) and three men (4%) selected the “don’t know” response when asked how dangerous they believed pythons to be.

Risk beliefs toward venomous snakes did not differ significantly between men and women; all but 2 of the 127 survey participants (98%) believed venomous snakes to be “very dangerous” and 91% (*n* = 116) considered themselves “very afraid” of venomous snakes.

### 3.3. Behavioral Responses

The most common response to seeing a python in the area around one’s home was to “do nothing” (*n* = 66, 52%). However, more women indicated that they would try to kill a python rather than “do nothing”, compared with only 10 (14%) men who reported trying to kill a python on sight rather than do nothing, X^2^ (1, *N* = 99) = 9.80, *p* = 0.002, V = 0.315). There was no difference between the reported behavior of men with experience on Tinjil Island and those with no experience on Tinjil Island when responding to seeing pythons in their neighborhood areas (Fisher exact test, *n* = 69, *p* = 0.70).

In response to encountering a venomous snake, 72% (*n* = 91) of respondents stated that their typical response would be to try to kill the snake. Only 2 individuals (2%) had actually killed a python in the past year, but 19 (15%) reported having killed at least one venomous snake in the same time period. Of these, four individuals claimed to have killed four or more venomous snakes in the past year.

The models of the behavioral responses when seeing both pythons and venomous snakes were significantly associated with respondents’ feelings when seeing snakes, and their desire for snake populations to go up or down (Figure 2, Table 2). Individuals with stronger negative feelings toward snakes and those with a desire for snake populations to decrease were more likely to attempt to kill a snake when encountering it.

## 4. Discussion

In our study area, the threat of SBE appears to be substantiated by the reported experiences. Three individuals had been bitten themselves in the previous year alone, and multiple first-hand accounts of past SBE or relatives who had been bitten by venomous snakes, including some fatal outcomes, were also documented by our survey. Attitudes and behaviors toward venomous snakes were generally consistent among the individuals surveyed, indicating commonly accepted beliefs within our study population that venomous snakes were both highly dangerous and undesirable in neighborhood areas. The significant positive relationship between knowledge of people bitten by venomous snakes and a desire for venomous snake populations to decrease demonstrates that a perceived threat of SBE may translate to negative attitudes toward snake conservation. Negative attitudes consistently predicted higher anti-conservation (i.e., attempt to kill) behaviors for those encountering both pythons and venomous snakes in their neighborhood areas.

Men with experiences on Tinjil Island responded more positively when asked about snake populations on Tinjil Island versus in their own neighborhood. Their responses to snakes were situation specific; in regard to an area where snakes could pose a threat to people, respondents wished for snake populations to decrease, but when responding about Tinjil Island, a natural protected wilderness area with no permanent human inhabitants, more responses indicated a desire for snake populations to either increase or stay the same. Experience on Tinjil Island did not translate to more conservation-leaning attitudes or behavior towards snakes in neighborhood areas.

Not surprisingly, the non-venomous reticulated python was considered less dangerous, eliciting fewer responses regarding the need to kill and/or to decrease populations of pythons in village areas. Women, and those with lower education levels, were more likely to have negative feelings when encountering pythons. Our findings are consistent with those of Pinheiro et al. [27], who, when surveying visitors to an urban scientific serpentarium in Brazil, also found women and those with lower levels of schooling to have more negative perceptions towards snakes. Differences between female and male responses also indicated that women were more fearful of pythons and were also more likely to attempt to kill them. Sixteen percent of our female survey participants responded “don’t know” regarding the danger posed by pythons, indicating a greater level of uncertainty about pythons as compared to venomous snakes, who were considered “very dangerous” by all but a single female respondent. Females were similarly associated with greater fear of snakes than males in a comparison of university student attitudes towards snakes in Turkey and Slovakia [28]. One possible explanation is that the female participants in our study, most of them housewives, may have had a comparatively lower level of familiarity with snakes than male participants, who were predominantly fisherman, and that this lack of experience could have led to a greater level of fear. Alves et al. [29] reported similar gender differences in attitudes towards snakes among a surveyed population of students in Paraiba State, Brazil. In both Alves et al. and the current study, the majority of respondents lived in a rural area where male-dominated occupations (e.g., fisherman, worker, farmer) increased the likelihood of snake encounters while the primary female occupations (e.g., housewife, merchant) occurred in locations where women would be less likely to encounter snakes.

Given these differences, conservation efforts may need to be tailored specifically to certain groups or situations; those who are fearful due to a lack of familiarity with snakes may respond well to becoming more knowledgeable, particularly in regard to non-venomous species. For example, Balakrishnan [30] documented a decrease in killings of the non-venomous Travancore wolf snake (*Lycodon travancoricus*), a Batesian mimic of the highly venomous Indian krait (*Bungarus caeruleus*), following the establishment of a multi-year public education program and citizen scientist network in Kerala, India, where both species are commonly found. The decrease in killing in the region was in part attributed to an improved ability of the general public to distinguish between the two species following the education program [30].

Whereas increasing understanding and awareness may help reduce unfounded fear and indiscriminate persecution of snakes, particularly non-venomous species mistaken for those presenting a threat of SBE, it can be argued that the attitudes and responses of men and women regarding venomous snakes were not significantly different because venomous snakes are universally considered to be dangerous in our study location. In such cases, anti-conservation behaviors toward venomous snakes may need to be addressed with a more pragmatic approach of discussing and applying practical mitigation options in high-risk areas. Accordingly, perhaps the most effective approach to venomous snake conservation in our study area would be to decrease the perceived threat of snakes to humans by focusing on reducing the number of encounters resulting in SBE and decreasing the severity of outcomes associated with SBE. In rural areas such as MB, where human–snake coexistence is often a necessity, understanding and disseminating information about the behavioral ecology and habits of local venomous snake species can guide species-specific mitigation efforts such as removing tall grass or relocating wood piles to limit snake attractants in the immediate residential area. The use of bed nets has also been associated with greater protection against SBE [31], and the distribution of personal protective equipment kits (including boots, flashlights, and mosquito nets) in Tirunelvi, Tamil Nadu [32] and the donation of beds, enabling Sri Lankan village recipients to sleep above the ground [33], have been implemented at the community level with positive outcomes in the reduction of SBE. Such approaches are relevant to village areas such as MB, where individuals are often barefoot or in sandals in areas where snakes may be present, and where sleeping on the floor is also common.

Once SBE has occurred, victims can be limited in their ability to access proper treatment quickly (e.g., this study, [34]). Even if a facility with antivenom is within reach, as of 2016, Indonesian medical facilities were only able to provide a single brand of polyvalent antivenom, Serum Anti Bisa Ular (SABU), produced by Biofarma, a state-owned enterprise [14,35,36]. This antivenom treats envenomation by neurotoxic species found in our study area, such as *N. sputatrix* and *C. rhodostoma*, but is not specifically formulated to cover the venoms of others (e.g., *Trimeresurus* spp.; [35]). SABU has also been shown to have less efficacy against the venoms of *N. sputatrix* and *Bungarus candidus* (Malayan krait) when compared to Thai Neuro Polyvalent Antivenom (NPAV) [36] and was markedly less potent than Thai *Ophiophagus hannah* monovalent antivenom (OHMAV) and Chinese *Naja atra* monovalent antivenom (NAMAV) when tested for reactivity against the venom of *O*. *hannah* [37]. Tan et al. [36] also identified impurities in SABU that could lead to an increased risk of hypersensitive reactions in individuals treated with this antivenom.

The production cost, storage, and distribution of antivenom pose yet additional difficulties in facilitating widespread availability. Given the difficulty of providing rapid access to antivenom and care in rural communities and noting traditional approaches to SBE mentioned by our survey participants and possible reluctance or inability to seek treatment at a formal health facility, further investigation into the efficacy of alternative remedies and plant-based medicinal treatments is also warranted (as in [38,39]). Whereas this study did not systematically assess respondents’ reasons for choosing traditional approaches over formal treatment (e.g., administration of antivenom at a medical facility), the limited effectiveness of SABU, coupled with the potential for adverse reactions, could potentially affect public confidence in the use of antivenom, further hindering efforts for improved SBE responses. Increasing the number of positive SBE outcomes will depend on multiple factors, such as heightened community awareness and acceptance of antivenom, improved antivenom, enhanced access, and availability of appropriate treatments.

## 5. Conclusions

The results of our study underscore the value of collecting survey data to both identify community needs, and to guide education and conservation efforts in achieving the greatest effect. Future steps should include systematic snake population surveys, and increased location-specific documentation of snake bites and snake bite treatments, to inform an increased local availability of appropriate SBE responses. An understanding of community attitudes towards antivenom, awareness of first aid protocols for SBE, and current treatment preferences among residents are also essential to guide local education and safety engagement efforts. Greater community knowledge of snakes and increases in local resources and preparedness can contribute equally to accomplish the goals of both snake conservation and improved public safety through prevention of SBE throughout southeast Asia.

## Figures and Tables

**Table 1:** Demographic profiles of survey participants (*N =* 127) interviewed about snake experiences, attitudes, and behaviors in Banten, Indonesia.

		Male *n* = 71 (56%)	Female *n* = 56 (44%)	Total *N* = 127
Age				
	19–30	3 (4%)	16 (29%)	19 (15%)
	31–45	40 (56%)	27 (48%)	67 (53%)
	46–60	23 (32%)	12 (21%)	35 (27%)
	61+	5 (7%)	1 (2%)	6 (5%)
Level of Education				
	None	9 (13%)	8 (14%)	17 (13%)
	Elementary School	43 (61%)	34 (61%)	77 (61%)
	Middle School/Junior High	11 (15%)	10 (18%)	21 (16%)
	High School	8 (11%)	3 (5%)	11 (9%)
	Post-Secondary	0	1 (2%)	1 (1%)
Primary Occupation				
	Housewife (*ibu rumah tangga*)	0	35 (62%)	35 (28%)
	Fisherman (*nelayan*)	32 (45%)	0	32 (25%)
	Merchant (*pedagang*)	4 (6%)	19 (34%)	23 (18%)
	Worker (*karyawan*)	12 (17%)	0	12 (9%)
	Entrepreneur (*wiraswasta*)	7 (10%)	0	7 (6%)
	Farmer (*petani*)	4 (6%)	0	4 (3%)
	Other	12 (17%)	2 (4%)	14 (11%)
Religion				
	Muslim	71 (100%)	56 (100%)	127 (100%)
	Other	0	0	0
Ethnic Group				
	Sundanese	55 (77%)	49 (88%)	104 (82%)
	Javanese	13 (18%)	5 (9%)	18 (14%)
	Indramayu	1 (<2%)	1 (<2%)	2 (1.5%)
	Lampung	1 (<2%)	1 (<2%)	2 (1.5%)
	Baduy	1 (<2%)	0	1 (1%)
Experience Working on Tinjil Island				
	No	40 (56%)	56 (100%)	96 (76%)
	Yes	31 (44%)	0	31 (24%)

**Table 2:** Explanatory variables of attitudes and behavior towards snakes based on cumulative link model model-averaging of top models with ΔAICc < 2. Model-averaged coefficients (β/Estimate) and standard error (SE) represent the strength of the predictors. Positive coefficients indicating more negative attitudes/behavior (e.g., hate seeing snakes/prefer snake populations to decrease/try to kill snakes). Significant predictors are highlighted in bold.

Feeling When Seeing Pythons					Feeling When Seeing Venomous Snakes				
	**Estimate**	**SE**	**Z**	**Pr (>|z|)**		**Estimate**	**SE**	**Z**	**Pr (>|z|)**
**Gender (male)**	**−1.53**	**0.36**	**4.21**	**2.55 × 10^−5^**	Education	−0.21	0.26	0.82	0.414
**Education**	**−0.49**	**0.22**	**2.19**	**0.028**	Bitten by snakes	0.67	0.56	1.19	0.233
Seeing snakes	−0.24	0.4	0.59	0.553	**Know people bitten**	**0.37**	**0.19**	**1.97**	**0.049**
					Seeing snakes	0.06	0.16	0.41	0.686
**Preferred python population trend**					**Preferred venomous snake population trend**				
	**Estimate**	**SE**	**Z**	**Pr (>|z|)**		**Estimate**	**SE**	**Z**	**Pr (>|z|)**
**Gender (male)**	**−1.54**	**0.36**	**4.25**	**2.15 × 10^−5^**	**Know people bitten**	**0.92**	**0.33**	**2.71**	**0.007**
Education	−0.36	0.27	1.31	0.189	Seeing snakes	0.15	0.46	0.32	0.746
**Behavior toward pythons**					**Behavior toward venomous snakes**				
	**Estimate**	**SE**	**Z**	**Pr (>|z|)**		**Estimate**	**SE**	**Z**	**Pr (>|z|)**
**Feeling**	**0.40**	**0.20**	**2.02**	**0.044**	**Feeling**	**1.01**	**0.29**	**3.43**	**0.001**
**Population trend**	**1.50**	**0.54**	**2.76**	**0.006**	**Population trend**	**1.43**	**0.63**	**2.27**	**0.024**
Fear of snakes	0.14	0.25	0.55	0.580	Fear of snakes	0.10	0.30	0.33	0.741

## Data Availability

The data associated with the manuscript are available upon formal request to the first author.
